# The critical role of platelet in cancer progression and metastasis

**DOI:** 10.1186/s40001-023-01342-w

**Published:** 2023-09-28

**Authors:** Lin Zhou, Zhe Zhang, Yizhou Tian, Zefei Li, Zhongliang Liu, Sibo Zhu

**Affiliations:** 1 Department of Microbiology, Immunology and Molecular Genetics, University of California, Los Angeles, CA 90095 USA; 2 Department of Gastrointestinal Surgery, Huizhou Municipal Central Hospital, Huizhou, Guangdong 516001 People’s Republic of China; 3 Department of Oncology, Zhoushan Hospital of Traditional Chinese Medicine (Affiliated to Zhejiang University of Traditional Chinese Medicine), Zhoushan, 316000 China; 4 School of Life Sciences, Fudan University, Shanghai, 200438 China

**Keywords:** Platelet, Metastasis, EMT, CTC

## Abstract

Platelets play a crucial role in cancer blood metastasis. Various cancer-related factors such as Toll-like receptors (TLRs), adenosine diphosphate (ADP) or extracellular matrix (ECM) can activate these small particles that function in hemostasis and thrombosis. Moreover, platelets induce Epithelial Mesenchymal Transition (EMT) to promote cancer progression and invasiveness. The activated platelets protect circulating tumor cells from immune surveillance and anoikis. They also mediate tumor cell arrest, extravasation and angiogenesis in distant organs through direct or indirect modulation, creating a metastatic microenvironment. This review summarizes the recent advances and progress of mechanisms in platelet activation and its interaction with cancer cells in metastasis.

## Introduction

Cancer metastasis is the cause of a huge proportion (around 90%) of cancer-related deaths, and curing metastatic diseases remains a challenge despite great progress in diagnosis of cancer. Many cancer types metastasize through the circulatory system. Metastasis begins when cancer cells in the primary tumor, enabled by processes such as the Epithelial Mesenchymal Transition (EMT), gain invasion power and spread to distant sites [1]. Cancer cells in the blood vessels, termed circulating tumor cells (CTCs), are confronted with several stress factors such as shear force, programmed cell death (known as anoikis), and attack from the immune system. Only a few CTCs will survive, adhere to the endothelium (usually of capillaries) at destination sites, and extravasate [2, 3].

Platelets are tiny anucleate cells that originate from megakaryocytes, and the process of platelet production takes place predominantly in the bone marrow. During platelet generation, long extensions of megakaryocytes called proplatelets grow into the vasculature and are broken off by blood forces. Proplatelets in the blood are further divided into smaller platelets in the circulatory system [4]. Traditionally, the main function of platelet is to participate in hemostasis and thrombosis. Yet, increasing evidence have suggested that platelets can perform a myriad of other functions. For instance, platelets express multiple functional Toll-like receptors (TLR) and can also secrete both pro-inflammatory and anti-inflammatory cytokines (how platelets regulate when to induce or suppress inflammation remains to be studied), thereby modulating immune system function [5, 6].

**Fig. 1:**
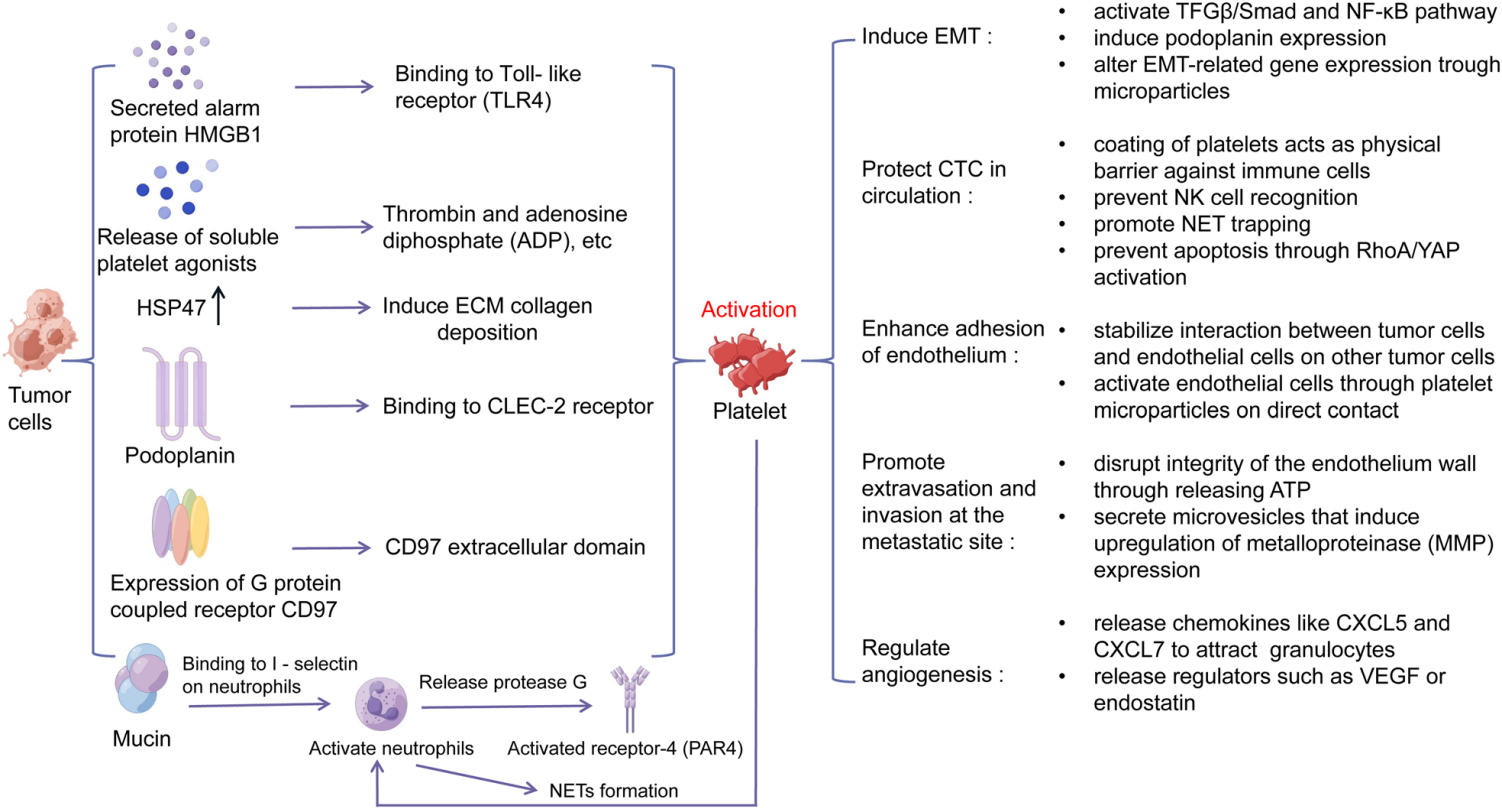
Activation of platelet by tumor further promotes the metastases of tumor in remote. Tumor cells activate platelets through various mechanisms, including secretion of the alarm protein HMGB1, release of soluble platelet agonists, induced increased expression of Hsp47, and expression of the G protein-coupled receptor CD97. Activated platelets induce EMT of tumor cells in indirect or direct ways by secreting microparticles (MPs), expressing various adhesion molecules and secreted growth factors, which further promotes the metastases of tumor in remote

As early as in 1865, Armand Trousseau has documented the development of venous thrombosis during the progress of pancreatic cancer, suggesting an interplay between cancer development and platelet activity and blood clot formation [7]. Since then, more clinical data have suggested an elevated risk of venous thromboembolism in cancer patients, and the risk depends upon factors including cancer type and stage [8]. Conversely, higher cancer morbidity is observed in patients with primary deep venous thromboembolism. In cancer patients, thrombosis is associated with poor prognosis such as increase in cancer progression and mortality [9]. Moreover, the association between platelet activity and cancer has been employed in clinical detection of cancer. Studies comparing platelet RNA profiles of healthy and metastatic non-small cell lung cancer (NSCLC) patients revealed 200 RNA transcripts with differing levels of expression [10]. Platelet RNA markers can even be leveraged to distinguish between patients with nonmetastatic and metastatic breast cancer [11]. Many studies have shown that platelets can directly bind CTCs from tumors of both epithelial (e.g., breast and lung) and mesenchymal (e.g., melanoma) origin. Using platelet markers to isolate platelet-cloaked CTCs resulted in higher CTC capture rates compared to traditional methods (EpCAM) of isolating CTCs [12]. Platelets’ diverse functions and their deep association with cancer progression prompt scientists to study platelets’ functions in cancer progression. Many studies have revealed new mechanisms of platelet interaction with the tumor and blood cells to aid cancer metastasis. In this review, we will summarize current findings on platelets’ role in cancer metastasis throughout different stages of metastatic development (Fig. 1).

### Tumor-induced platelet activation and aggregation

There is numerous evidence of tumor cells inducing platelet activation and aggregation. Activating platelets and forming a complex with them allow CTCs to harness the benefits of platelet’s promotion of tumor growth and metastasis. Activated platelets become adhesive and cluster around CTCs, protecting them against shear stress and attack from immune cells. Platelet interaction also allows CTCs to better adhere to vascular endothelium at the metastatic site. Activated platelets can also secrete a plethora of growth factors such as transforming growth factor beta (TGFβ), vascular endothelial growth factor (VEGF), and platelet-derived growth factor (PDGF) that promote pro-metastatic processes such as epithelial–mesenchymal transition (EMT) and angiogenesis.

Tumor cells can induce platelet activation and aggregation directly, either through release of soluble platelet agonists into the bloodstream or through contact between tumor cell and platelets. Tumor cells can release classic soluble factors such as adenosine diphosphate (ADP) [13]. The transmembrane protein podoplanin found on the surface of many cancer cell types can directly interact with platelet activation receptor CLEC-2. In a tumor environment, other cells could also be sources of podoplanin inducing platelet activation, such as vascular endothelial cells (VECs) and macrophages near the leaky vessels [14]. Besides, several cancer cell types express the G protein-coupled receptor (GPCR) CD97, and the extracellular domain of CD97 is also capable of binding and activating platelets [15]. Interestingly, tumor cells can also activate platelets via release of damage-associated molecular patterns. The alarmin protein HMGB1, which could be secreted by dying tumor cells in circulation, is a natural ligand of toll-like receptor 4 (TLR4) present on platelet surfaces [16]. Furthermore, Xiong et al. recently discovered that during EMT, human breast cancer CTCs can deposit the extracellular matrix (ECM) protein collagen, and Type I collagen present around CTCs is crucial for interaction with platelets and successful colonization. Under normal condition, platelets are only exposed to collagens during vascular injury, and collagen would induce platelet activation and blood clot formation under the scenario. Collagen deposition is induced through increased expression of Hsp47 in CTCs, which is a gene involved in ECM regulation [17].

Tumor cells can also indirectly activate and aggregate platelets through inducing pathways or formations of molecules that can lead to platelet activation. A common theme in many tumor cells’ mechanisms to activate platelets is releasing or inducing the expression of classic platelet agonists involved in the hemostasis or coagulation pathway. The tight association between platelet aggregating activity (PAA) and procoagulant activity (PCA) of tumor cells has been proposed in the twentieth century. PCA refers to the ability to convert prothrombin into the platelet agonist thrombin, which is a critical step in the blood coagulation pathway. As thrombin inhibitors can inhibit tumor cell-induced platelet aggregation by many tumor cell types, it is hypothesized that one-way tumor cells activate platelets is through activating coagulant factors, which eventually lead to the generation of thrombin. From rodent tumor cells, the protein with both PAA and PCA has been isolated. The component is termed cancer procoagulant (CP), and its activity is dependent on coagulant factor X but not VII. CP of tumor cells is thus proposed to be a factor X activator [18]. As coagulant factor X is a part of the extrinsic coagulation pathway while factor VII is involved in the intrinsic pathway, tumor cells seem to activate platelets through the extrinsic pathway. Few follow-up studies were done to confirm the identity and role of CP in cancer cell–platelet interaction. Besides CP, tumor cells also express tissue factors (TF). TF initiates the extrinsic coagulation pathway and is normally expressed by subendothelial cells. Therefore, TF is only exposed to circulation during vessel injury, and cancer cells can imitate vessel injury and induce platelet aggregation through surface expression of TF. Pancreatic tumors can also directly generate thrombin and induce platelet activity [13].

Tumor cells can also activate platelets indirectly via innate immune cells in circulation. Shao et al. have discovered that carcinoma mucins can bind to both P-selectins on platelets and L-selectins on neutrophils, bringing the two cell types close to each other. Through interaction with mucins as well as bidirectional signaling between platelets and neutrophils via P-selectin and P-selectin glycoprotein ligand-1 (PSGL-1), both platelets and neutrophils are activated. Eventually, cathepsin G is released from neutrophils, further inducing platelet activation and aggregation through protease-activated receptor-4 (PAR4) [19]. Moreover, neutrophil extracellular traps (NETs), which are externalized DNA and proteases from neutrophils, also contain the ability to activate platelets and induce thrombosis. There is a plenty of evidence of tumor-induced NET formation, which render it a highly relevant mechanism of tumor-induced platelet activation. Interestingly, platelets might also possess the ability to induce NET formation, again suggesting a self-perpetuating cycle of interaction between platelets and neutrophils [20].

### Induction of epithelial–mesenchymal transition (EMT)

During cancer metastasis, some cancer cells in the primary tumor gain invasion power, leave the primary tumor tissue, migrate into nearby stroma, and eventually intravasate into the blood or lymphatic system [1]. Platelets can induce this transition to a more invasive and resistant phenotype in tumor cells via inducing EMT. The critical steps of EMT in epithelial cells include cytoskeletal rearrangement and loss of epithelial polarity and tight cell–cell adhesion, which allows for greater motility and invasion capability [21, 22]. Epithelial cells undergoing EMT increase expression of N-cadherins and reduce level of E-cadherins on their surface, which repression mediates the detachment from epithelium. EMT also promotes cancer growth and metastasis through other ways. As some cancer epithelial tissue go through EMT, ECM proteins are also secreted and remodeled, forming a more favorable microenvironment for the tumor tissue [17, 21].

EMT in cancer cells can be induced by a plethora of signals including transforming growth factor β (TGFβ), which are stored and secreted by platelets. TGFβ secreted by platelets can induce EMT and subsequent metastasis in breast, colon carcinoma and ovarian cancer cells [23, 24]. Knocking out TGFβ in platelets led to largely lowered numbers of metastatic foci. TGFβ released by platelets induce TGFβ/Smad pathway in tumor cells, and direct interaction between platelets and tumor cells also activate the NF-κB pathway. The two pathways in tumor cells synergize to induce a pro-metastatic invasive mesenchymal phenotype in tumor cells [24]. Interaction with cancer cell podoplanin through CLEC-2 on platelets is another way through which platelets induce EMT in tumor cells. Besides destabilizing cadherin junctions, the integral membrane protein podoplanin can associate with proteins of the ezrin–radixin–moesin (ERM) family, which act as a link between actin cytoskeleton and podoplanin. Thus, podoplanin expression is sometimes associated with recruitment of ezrin to reorganize cytoskeleton. In several cell types, expression of podoplanin is correlated with impaired cell–cell adhesion and a more invasive phenotype [25]. Microparticles (MPs) secreted by platelets can also induce EMT in ovarian cancer cells. Upon internalization of MPs by ovarian cancer cells, the microRNA miR-939 in MPs will alter expression of EMT-related genes, causing reduction in levels of E-cadherin mRNA while increasing expression of mesenchyme-related genes like vimentin and fibronectin [26].

The location of where platelets promote EMT of tumor cells has also been studied, and it has been shown that platelets can induce EMT in both primary tumor cells and CTCs. It has been well established that platelets are capable of intravasating into tumors, and extravascular platelets have been imaged in the tumor microenvironment of many tumor cells [27]. In the primary tumor, platelet marker CD42b is significantly correlated with EMT markers such as increased Snail1 and reduction of E-cadherin, which supports that platelets promote EMT in the primary tumor [28]. Moreover, many studies have shown that platelets perform many other functions to promote tumor progression in the primary tumor microenvironment. There is evidence showing that platelets regulate vascular structure and maturation and promote tumor growth in the tumor microenvironment [27]. On the other hand, studies have revealed considerable variation in epithelial and mesenchymal features in circulating breast tumor cells. Some CTCs even contain primarily epithelial markers, justifying the possibility that platelets may further induce mesenchymal phenotypes in CTCs. Furthermore, platelet aggregation was detected in clusters of CTCs with mesenchymal phenotypes, and an increase in the number of CTCs that contain mesenchymal features is correlated with disease progression [29]. These results suggest a possible role of platelets in activating EMT and metastasis in CTCs.

### Protection of tumor cells in circulation

Tumor metastasis is a highly inefficient process, as most of the tumor cells in circulation die quickly after extravasation. Circulating tumor cells face many stressors, including programmed cell death (termed anoikis) upon detachment from the extracellular matrix, blood shear forces and attack from the immune system [2]. As a result, only a small proportion of CTCs will survive and successfully seed metastasis. Within circulation, the immune response against CTCs is strong, and many kinds of immune cells interact with CTCs to affect their survival and colonization. Natural killer (NK) cells contribute greatly to the elimination of CTCs. In mouse models, NK cell depletion by antibodies led to largely increased numbers of metastatic colony formations [30]. Activated platelets coat extravasated CTCs, and along with a mesh of fibrinogen, they formed a protective layer around CTCs against NK cell recognition and elimination. Interestingly, several studies found that when NK cell functions are disrupted in mouse models, depleting platelets or fibrinogen or blocking platelet activation have little effect on tumor metastasis [30, 31]. This suggests that a key mechanism through which platelets promote metastasis is helping tumor cells evade attack from immune cells, specifically NK cells.

Besides acting as a physical barrier between CTCs and NK cells, several other mechanisms of platelets preventing NK cell recognition and attack have been proposed. NK cells are activated through detecting either “missing self” or “induced self”. “Missing self” refers to low or absent expression of MHC class I protein whereas “induced self” refers to expression of ligands that can activate NK cell receptors such as natural killer group 2, member D (NKG2D) [32, 33]. Platelets target both recognition pathways. Coating of platelets causes NK cells to exhibit “pseudoexpression” of platelet markers like glycoprotein IIb and MHC class I. Since fusion of platelet and tumor cell membranes is observed, it is proposed that platelets transfer these surface molecules to tumor cells upon contact. Obtaining platelet MHC class I allows tumor cells to downregulate expression of their own MHC class I proteins to avoid recognition by T cells or NK cells through killer immunoglobulin-like receptors (KIR) mismatch, while preventing recognition through “missing self” pathway [34]. Besides, TGF-β secreted by active platelets reduced NKG2D levels on NK cells and release of antitumor cytokines like IFN-γ. This shows that platelets also target “induced self” recognition of tumor cells by NK cells [32]. Moreover, sheddases ADAM10 and ADAM17 contained in active platelet releasates induce shedding of NKG2D ligands like MICA and MICB on breast and colon tumor cell lines [33]. A more recent study by Cluxton et al. showed that when melanoma and ovarian cancer cells are cloaked by platelets, their surface expression of MICA and MICB also decreases significantly, accompanied by an increase in soluble MICA and MICB. However, in these cell lines, gene expression of ADAM19 rather than ADAM10 or ADAM17 is upregulated when the tumor cells are cloaked with platelets. The group also discovered a novel mechanism of how platelets help tumor cells evade NK cell recognition. Platelets downregulate CD226 and CD96 on NK cells, which are also involved in tumor cell clearing [35].

Besides NK cells, other immune cells can also interact with CTCs and influence the success of metastasis seeding, and platelets can regulate some of the interactions as well. Neutrophils can kill tumor cells through the production of hydrogen peroxide (H_2_O_2_), yet a greater neutrophil-to-lymphocyte ratio (NLR) correlates with the number of CTCs as well as metastasis of pancreatic ductal adenocarcinoma after surgery and poorer survival of primary breast cancer patients [36–38]. While neutrophils can clear CTCs via H_2_O_2_, some CTCs can develop resistance to neutrophil cytotoxicity and form clusters with neutrophils. The inflammatory interaction between CTCs and neutrophils promotes proliferation and dissemination of tumor cells. Neutrophils are also capable of infiltrating primary tumors, and primary tumors with mutations that grant resistance to neutrophil killing will shed more CTC-neutrophil clusters [38]. Platelets might also play a role in promoting neutrophil migration and inflammation. Neutrophils scan for activated platelets at an injured site in the vessel, and only when sufficient interactions occur between neutrophils and platelets do neutrophils organize receptors for migration across the vascular wall [39]. Nonetheless, the study was not performed under the context of tumors, and whether activated platelets provide crucial signals for neutrophils to infiltrate the primary tumor remains to be verified. Moreover, CTCs can also be trapped by NETs, which promotes CTC adhesion to the vasculature. Interestingly, in the mouse model of post-cancer surgery stress, coating of CTCs by platelets can promote NET trapping and subsequent metastasis formation [40]. Study has shown that CTC expression of programmed death-ligand 1 (PD-L1) can restrict T cell function and proliferation, yet details of the interactions are unknown [38]. One study showed that platelets suppress T cell proliferation and IFNγ production mainly through TGFβ on the cell-surface docking receptor GARP, which might contribute to the protection of tumor cells [41]. More study is required for us to understand how platelets mediate interactions between tumor cells and other types of immune cells.

**Fig. 2:**
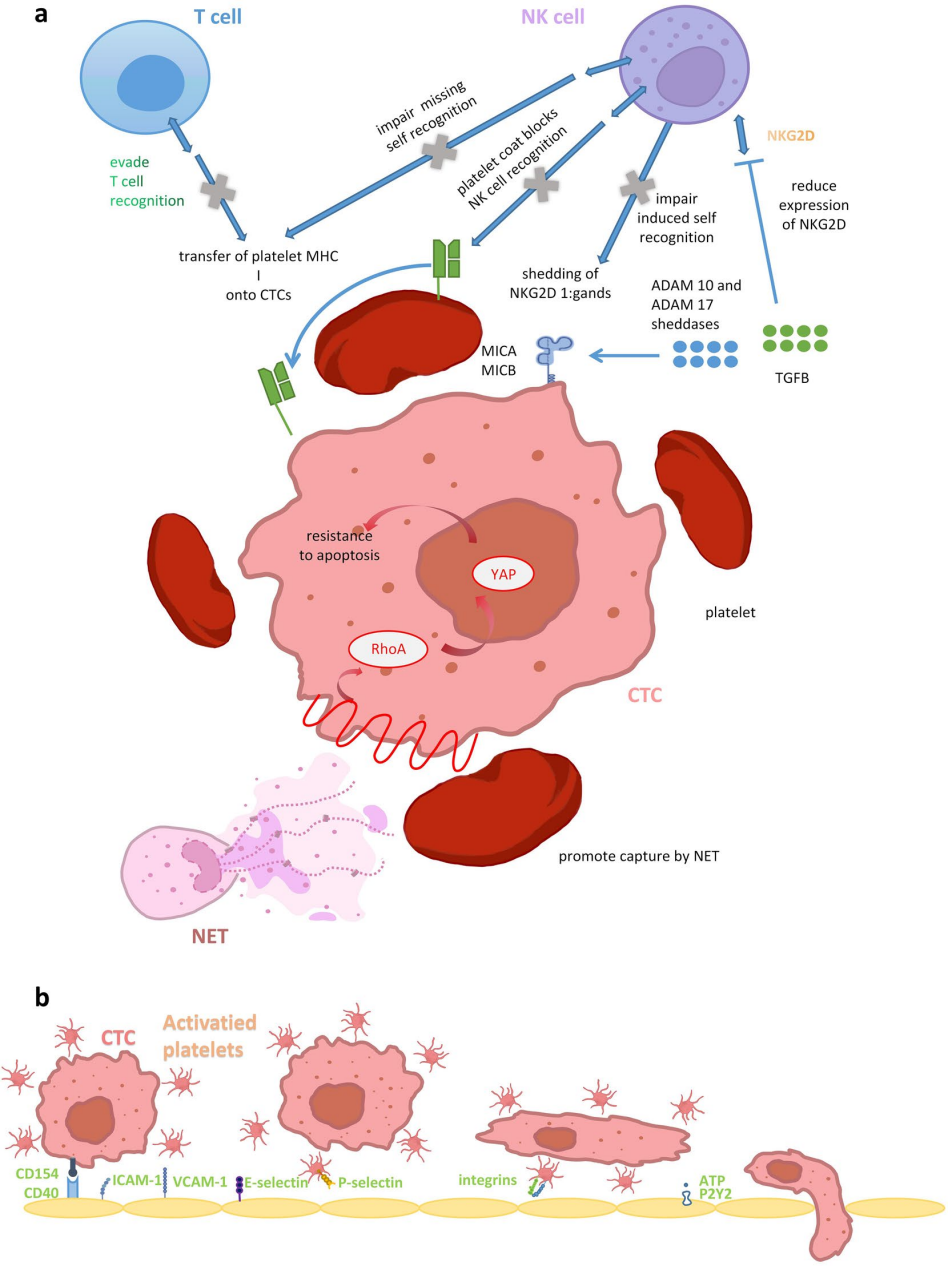
**a** Platelets protect CTCs within circulation. The platelet coat around CTCs physically shields them from NK cell attack. Moreover, platelets can transfer their MHC I molecules onto the membranes of CTCs. This allows CTCs to downregulate expression of their own MHC I molecules, evading T cell recognition without eliciting attack from NK cells through "missing self" recognition. Platelets can also impair NK cell recognition of CTCs via activation of NKG2D receptors. Platelets also promote association with NET to assist CTC distribution and adhesion. Through inducing the RhoA/YAP pathway, platelets promotes CTCs' resistance to apoptosis. **b** Through CD40/CD154 interaction or release of microparticles, platelets prime endothelid cells for attachment. Platelets promote capture of CTCs by the endothelium and reduce rolling rate. Mediated by integrins platelets-CTC complex adhere to the endothelium. ATP-released platelets interact P2Y2 receptor endothelid cells ceasing opening endothelid bar.

Besides evading immune attack, Haemmerle recently showed that platelets also promote metastasis by preventing anoikis in CTCs. Interaction with platelets induces RhoA activation and subsequent YAP activation, which leads to gene expression that confer tumor cells resistance to apoptosis [42]. Moreover, although providing resistance to shear stress and oxidative stress is a popular hypothesis regarding how platelets promote CTC survival, there is few evidence supporting it. Egan et al. have used lactate dehydrogenase release as a measurement for shear-induced membrane damage to show that CTCs are more resistant to shear stress under the presence of platelets [43]. Key events during platelets’ protection of tumor cells in circulation are depicted in Fig. 2a.

### Enhancing tumor cell adhesion to vascular wall at the metastatic site

Within circulation, CTCs quickly get trapped in the narrow capillaries, as their diameters are larger than those of the microvasculature. Nonetheless, there is evidence that CTCs can also arrest in wider vessels, and sustained attachment to the endothelium after rapid arrest varies among metastasis models [44]. These findings suggest that besides passive trapping in circulation, CTCs also form active and enduring adhesion with the endothelium to promote extravasation and seeding of metastatic foci.

Platelets also play a crucial role in assisting adhesion of CTCs to vasculature. Platelets express a wide range of adhesion molecules such as integrins (including αIIbβ3/CD41, αVβ3, α2β1, α6β1 and so on), immunoglobulin superfamily proteins (PECAM-1), C-type lectin receptor family proteins (P-selectin, CLEC-2), leucine-rich glycoproteins (GPIb-IX-V), etc. These molecules are capable of bridging connections between platelets and vascular wall, tumor cells, and other platelets [9, 27, 45]. Upon coating CTCs, platelets stabilize interactions between tumor cells and vascular endothelial cells and enhance adhesion efficiency [46, 47]. Moreover, Kunita et al. discovered that inducing platelet aggregation (by stimulating expression of Aggrus in tumor cells) promoted retention of circulating tumor cells and metastasis foci formation in experimental metastasis models [48]. Platelets likely enhance attachment to endothelium through “tethering/rolling” before a stable adhesion is formed, as platelet coating reduced the rolling rate of cancer cells on human vascular endothelial cells [9, 46]. It has also been suggested that activated platelets promote endothelial adhesion of colon carcinoma cells through a “secondary tethering mechanism” where incoming tumor cells bind to tumor cells already adhering to the vascular wall. P-selectin and αIIbβ3 are responsible for enhancing the secondary tethering process [49]. Platelets can also increase the binding capability of tumor cells through increasing the level of adhesion molecules on tumor cell surface. Carpinteiro et al. discovered that interaction between platelets and melanoma cells stimulates acid sphingomyelinase (ASM), which is responsible for inducing ceramide generation on the surface membrane of tumor cells. Ceramide can congregate and organize receptors and signaling molecules, and Carpinteiro’s team observed clustering of integrins α5 and β1 on tumor cells [50]. Furthermore, platelet can release and transfer integrin β3 chain to cancer cells, and platelet-derived microvesicles can transfer CD41 to tumor cells and increase their adhesion to fibrinogen and endothelial cells [47, 51].

Normally, platelets would only adhere to the endothelial wall when vascular injury occurred and materials in the extracellular matrix are exposed to blood elements. To enhance CTC binding to the vascular wall that are not experiencing significant damage, endothelial cells at the premetastatic site may be activated. Activation of endothelial cells can lead to their expression of adhesion molecules including E-selectin, P-selectin, vascular cell adhesion molecule (VCAM)-1 and intercellular adhesion molecule (ICAM)-1, all of which can facilitate tumor cell binding. Primary tumors can release soluble factors that induce activation of the endothelial cells at the metastatic site [44]. Besides tumor cells, platelets are also able to induce activation of vascular endothelial cells. Plantureux et al. recorded that microparticles released by platelets can induce expression of the adhesion molecule ICAM-1 by endothelial cells and subsequently enhance interaction between tumor cells and endothelial cells [47]. CD154 expressed by platelets can interact with CD40 on endothelial cells and upregulate a myriad of adhesion molecules such as E-selectin, P-selectin, ICAM-1 and VCAM-1 [52]. Moreover, Läubli’s team discovered that activation of human microvascular endothelial cells, as measured by expression of E-selectin, is observed only when both platelets and leukocytes are cocultured with tumor cells [53]. Several molecules known to be involved in platelets’ promotion of CTC arrest and adhesion are shown in Fig. 2b.

### Platelet promotion of extravasation and invasion at the metastatic site

After CTCs have stably adhered to the endothelium at metastatic site, platelets continue to facilitate the metastatic process, promoting transendothelial migration, invasion, and subsequent survival and proliferation of tumor cells. In an in vivo study, Weber showed that in thrombocytopenic mice, intravenously injected tumor cells mostly failed to extravasate after three days while most of tumor cells discovered in wild type mice had exited the vasculature. Moreover, activating integrin αVβ3 on tumor cells, which are crucial for promoting interaction with platelets, led to more efficient transendothelial migration in vitro [54]. These results highlighted the importance of platelet-tumor cell interaction in promoting tumor cell extravasation. One-way platelets promote migration through the endothelium barrier is disrupting the integrity of the endothelium wall. ATP released from dense granules within platelets interact with receptor P2Y2 on endothelial cells and promote gap formation in the endothelial barrier [55].

Platelets can also enhance the invasive ability of tumor cells through the extracellular matrix, and this is typically evaluated by measuring cell movement through the Matrigel layer in vitro. Pang’s team discovered that after stimulating mammary adenocarcinoma cells with activated platelet membranes and growing them on Matrigel, the degradation of Matrigel was enhanced. Platelet-tumor cell binding is necessary for this process as inhibiting both P-selectin and integrin αIIbβ3 abrogated platelet binding and tumor cells’ ability to degrade the ECM [56]. Interestingly, in some other tumor cell lines, microvesicles secreted by platelets alone can enhance tumor cell invasion. Janowska-Wieczorek et al. recorded that incubation with platelet-derived microvesicles induced upregulation of matrix metalloproteinase (MMP) expression by multiple lung cancer cell lines. MMPs have been shown to be crucial in cancer metastasis, and they also play a role in ECM degradation. Different tumor cell lines responded differently to platelet microvesicle stimulation, and highly metastatic cancer cell lines exhibited greater response to the stimulation, increasing expression of multiple transcripts of MMP [51].

Platelet and leukocyte interplay also strongly influence the success of seeding metastasis. It is believed that leukocytes generally have positive effects on promoting tumor cell survival at the metastatic site, and recruitment of white blood cells are correlated with metastasis. Studies showed that leukocytes usually exert their effects during early stages of metastasis, influencing the formation of new metastatic foci. For instance, while initial seeding of tumor cells (around 2 h after tumor cell injection) were unaffected, metastasis events were reduced during the first 48 h of intravenous colon carcinoma cell injection in mouse models with depleted granulocytes [57]. Similarly, blocking endothelial recruitment of monocytes through CCL5 have similar inhibitory effects on metastasis [53]. Leukocytes can be a significant source of metastasis-promoting molecules such as MMP-9 [58, 59]. Nonetheless, there is also evidence that suggests inhibitory effects of leukocytes on metastasis. Tumor-entrained neutrophils (TENs) in premetastatic lung niches have cytotoxic effects on incoming tumor cells through secretion of reactive oxygen species such as H_2_O_2_. Upon further inspection, G-CSF secretion may be responsible for neutrophil sequestration, but it is not sufficient to induce TEN cytotoxicity. CCL2 and CCL5 secreted by premetastatic lungs could be responsible for the entrainment of neutrophils [59]. Labelle concluded that the signals used to recruit and activate leukocytes, as well as the type of leukocytes involved, will lead to different effects on metastasis [44].

Several studies have focused on the effect of platelet-leukocyte interplay on tumor cell extravasation and survival. Platelets and granulocytes together form the premetastatic niche for injected colon carcinoma cells. In in vivo models of tumor metastasis, injected tumor cells are immediately coated by platelets, and activated platelets subsequently release chemokines CXCL5 and CXCL7, which are chemokines attracting granulocytes [57]. Many other processes through which platelets attract leukocytes and modulate immune responses have been proposed, yet the studies are done in other contexts such as vascular injury and inflammation. It is highly likely that platelets recruit white blood cells to the metastatic site with similar mechanisms, as inflammation and cancer are highly correlated. Investigating exactly how platelets recruit and activate leukocytes to influence metastatic success at the metastatic site can clarify platelets’ role in metastasis promotion.

### Platelet’s regulation of angiogenesis

Rapidly growing and dividing malignant cells demand plenty of nutrients and oxygen. As a result, angiogenesis, the formation of new blood vessels, is pivotal for cancer progression. It is required for tumors to break out of dormancy [60]. Platelets can secrete a huge variety of angiogenic regulators. Interestingly, some of the released regulators promote angiogenesis while others inhibit it. For instance, platelets store and secrete VEGF, bFGF and PDGF, which are crucial pro-angiogenic molecules, while endostatin and PF-4 are some of the main anti-angiogenic regulator contained in platelets [6, 61]. There is evidence that besides transporting and distributing angiogenic regulators originally generated by megakaryocytes, platelets selectively take up angiogenic molecules like VEGF and endostatin from the environment [62]. There are correlations between platelet counts and serum VEGF level in breast, colorectal, renal, and ovarian cancer patients, suggesting that platelets are a main source of angiogenic molecules in circulation [63]. Furthermore, platelet depletion in metastatic melanoma models led to reduced vessel density in the tumor environment [64]. Incubation of releasates from activated platelets with human umbilical vein endothelial cells (HUVECs) led to increased endothelial cell tube formation [65]. These results again verified a generally positive role of platelets in promoting angiogenesis, and they even suggested that platelets can enhance the angiogenic potential of tumor cells.

Since platelets contain both pro-angiogenic and anti-angiogenic molecules, the mechanisms by which platelets regulate the release of these counteracting signaling molecules has been an area of active research. The specificity of signaling molecules and receptors seem to play a huge role on platelet’s effect on angiogenesis. Previous studies suggest that stimulation of PAR1 on platelet surfaces leads to release of pro-angiogenic molecules like VEGF and inhibits secretion of anti-angiogenic molecules like endostatin, while stimulation of PAR4 induces secretion endostatin and inhibits VEGF release [66]. Interestingly, incubation of platelets with the classic platelet agonist thrombin, which can activate both PAR1 and PAR4, led to little release of VEGF or endostatin, which could be explained by it activating both arms of the counteracting pathways [62, 66]. However, further studies showed that platelet releasates under both PAR1- and PAR4- stimulation promote angiogenesis, and PAR1-stimulated releasates exhibited a greater effect [9]. This is in line with previous conclusions that PAR-4 stimulation inhibited platelets’ effects on angiogenesis, although its effect might not be strong enough to overpower platelets’ overall positive effect on angiogenesis. Jiang et al. further demonstrated that co-incubating HUVECs with breast cancer cells can also promote endothelial tube formation, and both PAR1- and PAR4- induced platelet releasates can enhance the angiogenic potential of cancer cells (agreeing with previous results, PAR1-induced platelet releasates still exert a stronger effect) [65]. Nonetheless, the exact mechanism of how platelets regulate release of thematically different regulators has not been fully elucidated, and the regulation of angiogenic molecule release by platelets is not as simple and straightforward as some previous studies have proposed. It has been speculated that pro-angiogenic and anti-angiogenic regulators are stored in distinct α-granules within the platelet, and different signals would induce thematic release of certain subsets of granules [9]. Nonetheless, a study employing super-resolution immunofluorescence found that some α-granules may contain molecules with antagonistic function, and the distribution of these molecules within granules is heterogeneous and segregated. In the future, studies about the storage of signaling molecules in platelets and individual granules, the process of releasing these molecules (for instance, routes of α-granules towards the open canalicular system (OCS) and plasma membrane) and the interplay between different signaling pathways in platelets can all better our understanding of how platelets regulate angiogenesis under different scenarios [67].

Platelet-derived extracellular vesicles (PEV) can also induce angiogenesis upon transfer into vascular endothelial cells [68]. Stimulation of A549 lung cancer cells with platelet-derived microparticles, which are a category of PEVs, led to increased transcription of genes that code for angiogenesis-promoting molecules such as VEGF, HGF, and IL-8 [51]. Brill et al. showed that inhibiting VEGF, bFGF or PDGF all led to decrease in vessel formation promoted by PMPs [61]. In a recent study conducted by Anene et al., PEVs can deliver microRNA Let-7a to HUVECs, which targets the expression of anti-angiogenic factor THBS-1 [69]. The variations of angiogenic regulator levels in platelets and platelet releasates highlight their role in regulating angiogenesis, which is tightly linked to cancer progression.

### Inhibition and prevention of cancer metastasis by antiplatelet (AP) drugs

Given increasing evidence for platelets’ promotion of cancer metastasis, platelets are becoming increasingly popular as targets for preventing or attenuating metastasis. The effect of the classic AP drug aspirin has been a research hotspot, and multiple clinical and in vivo studies have supported its role in reducing metastasis in a wide range of cancer types. In a in vivo study using mouse models with induced highly metastatic hepatocellular carcinoma, treatment with high dose aspirin (0.5%) significantly reduced degree of metastases [70]. In a clinical analysis of prostate cancer patients treated with radiotherapy, patients taking aspirin exhibited reduced distant metastases rate (DMR) [71]. Triple-negative breast cancer patients who have taken AP drugs, which include aspirin and clopidogrel, exhibited reduced DMR and improved disease-free survival [72]. In a prospective observational study, among nurses diagnosed with breast cancer, those who took aspirin more than one day per week showed lower risk of cancer-related death [73]. Currently, more clinical trials are underway to investigate the effect of aspirin on metastasis and survival in other cancer types, such as colorectal and nasopharyngeal cancer [74].

Aspirin belongs to the class of non-steroidal anti-inflammatory drugs (NSAIDs). Aspirin can inhibit two isoforms of cyclooxygenase (COX), which are responsible for the conversion of arachidonate to prostaglandin G2 and subsequent production of prostaglandin H2 [75]. These prostaglandins are precursors of several functional prostanoids [76]. The COX-2 isoform is overexpressed in multiple types of cancer cells, and it plays a role in the generation of PGE2, which promotes tumorigenesis and metastasis. The COX-1 isoform is expressed by platelets, and it is responsible for the synthesis of TxA2 [77]. TxA2 is secreted by activated platelets, and it acts in an autocrine or paracrine manner to further induce platelet activation and aggregation [78]. Aspirin irreversibly inhibits COX-1 activity through acetylation of its serine residue 530, making the active site inaccessible for arachidonate [75]. As a result, defect in TxA2 production by platelets will impair platelet activation and negatively impact their promotion of cancer metastasis. Disruption of platelet activation is an ideal target for AP drugs to prevent or reduce cancer metastasis. As reviewed above, platelets impact cancer metastasis in a multi-faceted manner, involving many different mechanisms such as EMT, angiogenesis and evasion of host immune attack, and a large proportion of these mechanisms require platelet activation. Instead of targeting a single pathway, targeting platelet activation can lead to subsequent inhibition of multiple pathways promoting cancer metastasis. One in vitro study demonstrated that aspirin can reduce the ability of platelets to promote cancer cell invasion and induce EMT, both of which contributes to cancer metastasis [79].

While a body of observational and animal studies point to the effects of using aspirin in cancer therapy, more randomized clinical trials are required to verify their anti-tumor function. Several aspects of using aspirin as a cancer treatment remain to be studied. It is unknown whether aspirin along with other cancer treatment options is more effective compared to using aspirin alone. Specific mutations and markers that make certain patient populations more responsive to aspirin treatment have not been identified. Moreover, aspirin use has side effects that must be taken into account, especially damage to the gastrointestinal tract [74].

Besides aspirin, many other drugs targeting platelets have been proposed to have potential for reducing cancer metastasis. It is worth noting that most of these drugs target processes involved in platelet activation and TCIPA. A 5-nitrobenzoate compound 2CP can bind CLEC-2, which disrupts podoplanin-CLEC-2 interaction and subsequent TCIPA. In vivo, 2CP can reduce the number of metastasis foci in mouse glioma models [80]. The GPIIb/IIIa inhibitor XV454 significantly reduces metastasis in mouse models. GPIIb/IIIa is involved in stimulation of TCIPA by thrombin [81]. The ADP receptor P2Y12 inhibitor clopidogrel are already prescribed to cancer patients [77]. Interestingly, tamoxifen, the classic antiestrogen drug for breast cancer treatment, and its metabolite 4-hydroxytamoxifen can also inhibit tumor cell-induced platelet activation and lower the ability of platelets to produce VEGF and promote angiogenesis [82]. Moreover, platelets can also be utilized as drug delivery options. They can protect drugs in circulation for a prolonged period of time, and they are an effective way to target CTCs due to their extensive interactions [83].

Although targeting platelets seems promising to impair cancer progression, there are several challenges of targeting platelets in cancer treatment. The biggest challenge is the versatile functions of platelets. As mentioned above, platelets can have pro-angiogenic or anti-angiogenic and pro-inflammatory or anti-inflammatory effects, and we still have very limited understanding of the conditions that stimulate the different platelet functions. As a result, it is difficult to predict the exact consequences a drug blocking platelet activation will have. Moreover, depending on the dose and patient conditions, the same drug can have different effects. Overall, many more basic and clinical research is required for us to benefit from targeting platelets in battling cancer [77, 84].

## Discussion/conclusion

Despite a few studies pointing at platelets’ role in inhibiting cancer metastasis, overwhelming evidence suggests that platelets play a generally positive role in promoting cancer metastasis. Platelets play a role in almost all processes of cancer metastasis, from promoting EMT in the primary tumor, to protecting CTCs from immune attack and shear stress, and eventually assisting CTC adhesion and extravasation at the metastatic site (Fig. 2). Interestingly, a lot of mechanisms through which platelets affect metastasis does not involve molecules or processes seen in hemostasis or the coagulation cascade.

The positive effects of platelets on metastasis have made them new targets for preventing or attenuating metastasis in cancer patients. Nonetheless, one challenge is that due to platelet’s diverse functions, targeting one pathway involved in platelet’s promotion of metastasis may only have limited effects. However, as many pathways require platelet activation and aggregation, targeting platelet activation and binding may inhibit many pathways used by platelets to promote metastasis. Activated platelets secrete contents in the intracellular granules that contain growth factors such as VEGF and TGFβ, which promote processes such as angiogenesis and EMT in tumor cells. Furthermore, coating of platelets around CTCs following platelet activation protects CTCs from immune attack and shear stress and favors adhesion to the metastatic site. As reviewed above, drugs such as aspirin, which inhibits synthesis of platelet agonist TxA2, shows promising effect on reducing metastasis rate in animal models and clinical trials.

Nonetheless, there is still one complication regarding inhibition of platelet activation. Although activation by signals including CLEC-2, ADP and thrombin all lead to key platelet activation pathways such as increase of integrin receptor expression, certain platelet functions may depend upon signaling through certain platelet agonists [85]. For instance, processes such as regulation of vascular permeability and development depend upon signaling through receptors containing the immunoreceptor tyrosine-based activation motif (ITAM) sequence, such as CLEC-2, rather than through G protein-coupled receptors (GPCR), which includes receptors for ADP and thrombin. Thus, blocking different pathways in platelet activation can lead to different effects on cancer metastasis.

Study of platelets’ effects on metastasis continues to expand our understanding of the diversity of platelet functions, and there are still many unanswered questions about the mechanisms through which platelets affect metastasis. New findings made in the future can lead us to more effective ways to combat cancer metastasis.

## Data Availability

Not applicable.
